# The effect of IFN-γ and TGF-β in the functional activity of mononuclear cells in the presence of *Entamoeba histolytica*

**DOI:** 10.1186/s13071-015-1028-6

**Published:** 2015-08-08

**Authors:** Lucélia Campelo Albuquerque Moraes, Eduardo Luzía França, Rafael Souza Pessoa, Danny Laura Gomes Fagundes, Mara Gil Hernandes, Victor Pena Ribeiro, Maria Aparecida Gomes, Adenilda Cristina Honorio-França

**Affiliations:** Department of Parasitology, Institute of Biological Sciences, Federal University of Minas Gerais, Belo Horizonte, Minas Gerais Brazil; Institute of Biological and Health Science, Federal University of Mato Grosso, Rodovia BR070, Km 5 s/no, Barra do Garças, MT Brazil

**Keywords:** Leukophagocytosis, Amoebicide activity, Cytokines, MN cells, *Entamoeba histolytica*

## Abstract

**Background:**

*Entamoeba histolytica* (*E. histolytica)* causes amoebiasis, which is a disease with significant morbidity and mortality. Phagocytic cells and cytokines appear to be important in amoebiasis, but very little is known about the influence of these cells and cytokines in protozoan infections. The aim of this study was to analyse the supernatant of cultures of mononuclear (MN) cells with *E. histolytica* to determine: 1) the levels of the cytokines IFN-γ and TGF-β, and 2) the amoebicidal activity of MN cells after incubation with cytokines.

**Methods:**

Blood samples were collected from 30 volunteer donors. The cytokine concentrations in MN cells culture supernatants, superoxide release, leukophagocytosis, amoebicide activity, intracellular calcium release and apoptosis were analysed.

**Results:**

The IFN-γ concentrations were 6.22 ± 0.36 and TGF-β concentrations were 17.01 ± 2.21 in cells–trophozoite culture supernatants. MN cells, independently of cytokines, in the presence of amoeba increase the superoxide release. In the absence of cytokines, the ingestion of MN cells by amoebae was higher. In the presence of IFN- γ or TGF- β, a lower ingestion of MN cells was observed by amoebae. MN cells treated with cytokines exhibited higher amoebicide and apoptosis indexes. The incubation of cytokines increased the intracellular calcium release by MN cells.

**Conclusions:**

These results suggest that cytokines play a beneficial role for the host by activating MN cells against *E. histolytica*. The increased death of amoebae during the leukophagocytosis suggests that both cytokines (IFN-γ and TGF-β) can modulate the functional activity of MN cells and that these cytokines probably are important in the control of amoebic infections.

## Background

Amoebiasis is considered the second leading cause of death worldwide due to parasitic diseases, causing approximately 40 to 100 000 deaths per year [1]. The aetiologic agent is a protozoan, *Entamoeba histolytica* (*E. histolytica*) that colonizes human intestines and presents two evolutionary forms: the cyst and trophozoite [2]. Infections are usually asymptomatic, but in approximately 10 % of cases, the trophozoites penetrate the gut tissue, initiate hemorrhagic colitis and induce amoebic liver abscess [3].

Acute amoebiasis lesions are characterized by the presence of inflammatory cells that are recruited by proinflammatory signals produced by epithelial cells and other host cells. Proinflammatory cytokines are responsible for the recruitment of neutrophils [4] and the release of inflammatory mediators that promote the migration of neutrophils and macrophages [5, 6].

Neutrophils and macrophages are immune cells involved in phagocytosis, which serve as a major mechanism for the destruction of microorganisms beginning with the adhesion of these immune cells to the cell membrane. This interaction can be enhanced by hormonal and immunological factors [7–12].

However, few studies have evaluated the interaction between amoebae and phagocytes. It is known that *E. histolytica* is capable of inhibiting the production of active oxygen metabolites by monocytes [9], which probably prevent death of the parasite during leukophagocytosis.

The identification of mediators involved in leukocyte activation during infection by *E. histolytica* are of fundamental importance for understanding host responses in amoebiasis. Cellular interactions and cytokines have been reported during amoebic infections, and cytokines have been shown to be able to regulate monocyte function and increase the amoebicidal activity of monocytes [13–15].

Experimental studies have demonstrated that macrophages isolated from liver abscesses are refractory to activation by IFN-γ [16]. The anergy of these cells appears to be related to the suppression of Th1 cytokine production [TNF-α and IFN-γ], without interfering with the production of Th2 cytokines [IL-4 and IL-5]. IFN-γ and TGF-β appear to be important for the activation of macrophages and the destruction of *E. histolytica* [17]*.*

Despite advances in studies that have demonstrated the pathophysiological mechanisms of amoebiasis, the role of cellular immunity and their interactions with cytokines in this disease is unclear. The present study analysed the supernatant of cultures of MN cells with *E. histolytica* to determine: 1) the levels of the cytokines IFN-γ and TGF-β; and 2) the amoebicidal activity of MN cells after incubation with cytokines.

## Methods

### Ethics statement

This study was approved by the Institutional Research Ethics Committee of Araguaia University Center, and all of the subjects gave written informed consent before entering the experimental protocol.

### Blood sampling and MN cell separation

Blood samples (10 mL) were collected from 30 volunteer donors in tubes with anticoagulant. The samples were centrifuged at 160×*g* for 15 min to separate the plasma from the cells. Cells were separated over a Ficoll-Paque gradient (Pharmacia, Uppsala, Sweden); producing preparations of 95 % MN cells as analysed by light microscopy. MN cells were resuspended independently in serum-free 199 medium at a final concentration of 2 × 10^6^ cells mL^−1^. The MN cells were used immediately for superoxide release, leukophagocytosis, amoebicide activity, intracellular calcium release and apoptosis assays.

### *Entamoeba histolytica* strain

Trophozoites of the virulent strain of *E. histolytica* HM1:IMSS were grown axenically in a TYI-S-33 medium. Parasites were maintained with thrice-weekly sub-cultures, assuring their use during the exponential growth phase [9].

Amoebas from axenic cultures were centrifuged at 200×*g* in individual tubes, washed twice in PBS (*phosphate buffered saline –* pH 7.2) and adjusted to 4 × 10^4^ amoebae/mL.

### Cultures of MN cells and E. histolytica

After separation MN cells were centrifuged and the resuspended in RPMI culture medium supplemented with 10 % fetal bovine serum. The cells (2 × 10^6^cells/mL) were incubated with *E. histolytica* (4 × 10^4^ parasites/mL) for 2 at 37 °C with 5 % CO_2_. After this period, the cultures were centrifuged for 10 min at 160 × g, and the supernatant was reserved for cytokine quantification.

### Cytokine detection by ELISA (Enzyme Linked Immunosorbent Assay)

IFN-γ concentrations in the supernatant of cultures of MN cells with *E. histolytica* were determined by an ELISA kit from BioLegend® Legend Max™([San Diego, USA), and TGF-β concentrations were analysed using an ELISA kit from Enzo® Life Sciences (United Kingdom). The reaction rates were measured by absorbance in a spectrophotometer with a 450 nm filter. The results were calculated using the standard curve and shown in pg/dL.

### Treatment of MN cells with cytokines

To assess the effect of cytokines (IFN-γ or TGF-β) on superoxide anion release, leukophagocytosis, amoebicidal activity, intracellular calcium release and apoptosis, MN cells (2×10^6^ cells/mL) were incubated with IFN-γ or TGF-β at concentration of 100 ng/mL (Sigma ST Louis, USA,) [18] for 1 h at 37 °C. The MN cells were then washed once with 199 medium at 4 °C and immediately used in the assays. A control was performed with only 199 medium.

### Release of superoxide anion

Superoxide release was determined by cytochrome C (Sigma, ST Louis, USA) reduction [19]. Briefly, MN cells and *E. histolytica* trophozoites were mixed at a ratio of 1:2 and incubated for 2 h for leukophagocytosis. The suspensions (MN cells and amoeba) were then resuspended in PBS containing 2.6 mM CaCl2, 2 mM MgCl2, and cytochrome C (Sigma, ST Louis, USA;2 mg/mL). The suspensions (100 μL) were incubated for 60 min at 37 °C on culture plates. The reaction rates were measured by absorbance at 550 nm, and the results were expressed as nmol/O^2−^. All experiments were performed in duplicate.

### Cellular viability

Cellular viability was evaluated using the acridine orange method [10]. Cells were pre-treated with cytokines as described previously [18] and resuspended in serum-free 199 medium and centrifuged. The supernatant was discarded, and the sediment was dyed with 200 μL acridine orange [Sigma, ST Louis, USA; 14.4 g/L] for 1 min. The sediment was resuspended in cold 199 medium, washed twice and observed by immunofluorescence microscopy at 400x and 1000x magnification. The viability index was calculated by counting the number of orange- stained [dead] and green- stained [alive] cells out of 100.

### Amoebicide assay

Leukophagocytosis and microbicidal activity were evaluated by the acridine orange (Acros organics, New Jesse, USA) method [10]. Equal volumes of parasite (4×10^4^ parasites/mL) and MN cell (2×10^6^ cells/mL) pre-treatment or not with cytokines were incubated at 37 °C for 2 h under continuous shaking. Leukophagocytosis was stopped by incubation on ice. The suspensions were centrifuged twice (160×*g*, 10 min, 4 °C). The suspension was resuspended in serum-free 199 medium and centrifuged. The supernatant was discarded, and the sediment dyed with 200 μL of acridine orange (Sigma, ST Louis, USA; 14.4 g/L) for 1 min. The sediment was resuspended in cold 199 medium and washed twice. Cellular viability, phagocytosis and death of trophozoites and MN cells were determined by fluorescence microscopy at 400× and 1000× magnification. One hundred amoebas were counted per slide.

Leukophagocytosis was considered positive when the trophozoite contained internalized MN cells. The amoebicide index is calculated as the ratio between orange-stained (dead) and green- stained (live) amoeba × 100 [10]. All experiments were performed in duplicate.

### Apoptosis assay

Annexin V staining was used to assess apoptosis. Untreated cells were used as negative control and cells treated with staurosporin [Sigma ST Louis, USA −20] were used to induce apoptosis, as positive control. Controls and MN cells treated with cytokines and incubated with *E. histolytica* were resuspended in 500 μL of binding buffer containing 5 μL of annexin V-FITC (Annexin V-FITC Apoptosis Detection Kit, Alexis TM, San Diego, USA) and then incubated for 10 min. at room temperature. Fluorescence of the cells was analyzsed by flow cytometry (FACS Calibur system - BD, San Jose, USA).

### Intracellular Ca^2+^ release determination

We performed fluorescence staining on the FACS Calibur (BD San Jose, USA) to assess intracellular Ca^2+^ release in MN cells [21]. Cells were loaded with the fluorescent radiometric calcium indicator Fluo3-Acetoxymethyl (Fluo3-AM– Sigma ST Louis, USA). Cell suspensions, pre-treated or not with 5 μL of cytokines (Sigma, final concentration of 100 ng/mL), were mixed and incubated at 37 °C for 30 min under continuous stirring. Suspensions were centrifuged twice (160×*g*, 10 min, 4 °C) and resuspended in PBS containing BSA (5 mg/mL). This suspension was incubated with 5 μL of Fluo-3 (1 μg/mL) for 30 min at 37 °C. After incubation, MN cells were washed twice in PBS containing BSA (5 mg/mL; 160×*g*, 10 min, 4 °C) and then analysed by flow cytometry (FACS Calibur system - BD, San Jose, USA). Fluo-3 was detected at 530/30 nm filter for intracellular Ca^2+^. The rate of intracellular Ca^2+^ release was expressed as the geometric mean fluorescence intensity of Fluo-3.

### Statistical analysis

Data were expressed as the mean ± standard deviation (SD). Using analysis of variance (ANOVA), statistically significant differences were evaluated for the superoxide release anion, phagocytosis, amoebicide index and intracellular Ca2+ release in the presence or absence of cytokines, and the differences were considered statistically significant for p-values less than 0.05.

## Results

The IFN-γ (pg/mL) and TGF-β (pg/mL) concentrations in supernatant in cultures of MN cells were 2.10 ± 0.62 and 2.60 ± 0.82 respectively. In supernatant in cultures of MN cells and *E. histolytica* the cytokine concentrations were 6.22 ± 0.36 for IFN-γ and 17.01 ± 2.21 for TGF-β (*P* < 0.05). The viability of MN cells and amoeba in the presence or absence of cytokines is shown in Table 1. The IFN-γ and TGF-β did not alter the viability of MN cells.

**Table 1:** Mean (±SD) of MN cell viability and *E. histolytica* viability in the presence of cytokines (INF-γ or TGF-β)

Viability (%)	
MN cells	96.4 ± 2.0
MN + INF-γ	89.2 ± 3.7
MN+ TGF-β	89.0 ± 4.2
*E. histolytica*	95.7 ± 1.7
*E. histolytica* + INF-γ	94.2 ± 3.8
*E. histolytica* + TGF-β	89.1 ± 4.7

The results are presented as mean and standard deviation. *P* > 0.05

MN cells, independently of cytokines, showed the highest superoxide release when exposed to the parasite when compared with the spontaneous superoxide [Table 2].

**Table 2:** Superoxide release by MN cells at different incubation times (mean ± SD, *N* = 10 in each treatment)

Groups	Superoxide Release (nmol)
MN Cells	3.6 ± 0.4
MN Cells + *E.histolytica*	8.6 ± 0.5^a^
MN Cells *+ E. histolytica* + IFN-γ	9.3 ± 0.9^a^
MN Cells *+ E. histolytica* + TGF-β	8.2 ± 0.8^a^

MN cells were treated with cytokines or untreated, in the presence or absence of *E. histolytica*

^a^Indicates statistically significant differences between MN cells treated with cytokines or left untreated and incubated with parasites and the control (without parasite)

**Fig. 1:**
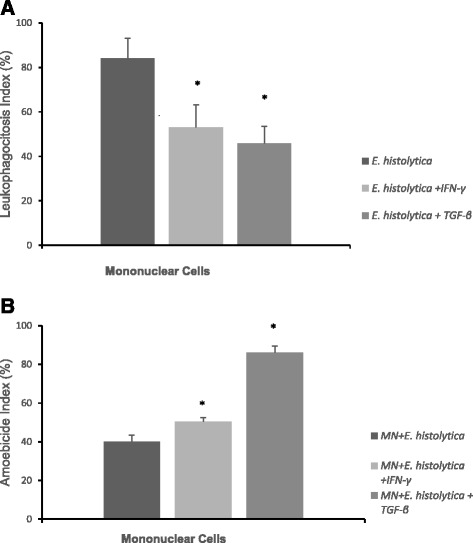
Leukophagocytosis (**a**) and Amoebicide index (**b**) by MN cells (mean ± SD, *N* = 10 in each treatment), determined by the acridine orange method. MN cells were incubated with *Entamoeba histolytica* (*E. histolytica*) in the presence of gamma interferon (IFN-γ) and transforming growth factor β (TGF-β). *indicates statistically significant differences from the 199 medium and cytokines

An analysis of the ingestion of MN cells by amoebae in the absence of cytokines revealed a higher phagocytosis index after 2 h of incubation. In the presence of cytokines, a lower ingestion of MN cells by amoebae was observed. No difference in the ingestion of MN cells by the amoebae were observed for cytokines IFN-γ and TGF-β [Fig. 1a].

In general, in the absence of cytokines, a low percentage of dead amoebae were observed during MN cell internalization. In the presence of cytokines, an increase in the percentage of dead amoebae during MN cell internalization was observed. MN cells treated with cytokines exhibited a higher amoebicide index. The highest amoebicidal activity was observed in MN cells treated with TGF-β [Fig. 1b].

**Fig. 2:**
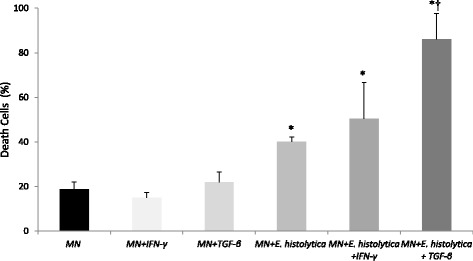
Apoptosis by Annexin-V assay in MN cells and *E. histolytica*. Results are expressed as the mean and standard error of six independent experiments. MN cells were incubated with *Entamoeba histolytica* (*E. histolytica*) in the presence of gamma interferon (IFN-γ) and transforming growth factor β (TGF-β). *indicates statistically significant differences between MN cells incubated with cytokines and *E. histolytica* and the control (only MN cells); † indicates statistically significant differences between MN cells incubated with cytokines and *E. histolytica* and the MN cells without cytokines and *E. histolytica*

**Fig. 3:**
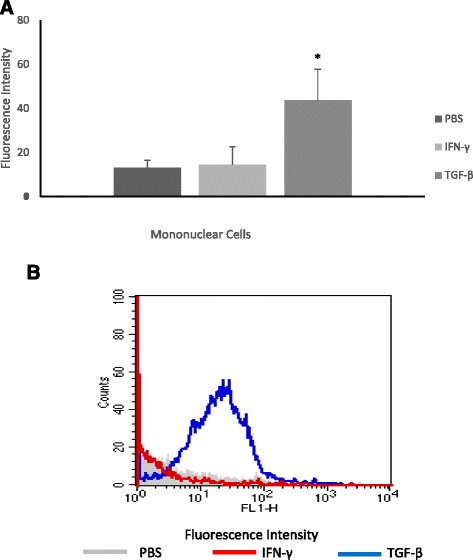
Intracellular Ca2+ release (**a**) by mononuclear (MN) cells indicated by geometric mean fluorescence intensity of Fluo-3. MN cells were pre-incubated with cytokines or left untreated. Intracellular Ca2+ release (**b**) after 2 h of incubations. *indicates statistically significant differences between MN cells incubated with cytokines and the control [PBS]. Cells were stained with Fluo-3 [Fluo3-Acetoxymethyl], and immunofluorescence analyses were carried out by flow cytometry [FACScalibur, Becton Dickinson, USA]

To evaluate the apoptosis induced by the interaction of MN cells and amoeba, the annexin V assay was performed by flow cytometry [Fig. 3]. The IFN-γ and TGF-β induce low apoptosis indexes in MN cells. The interaction of MN cells and amoeba showed death of these cells. In the presence of cytokines, an increase in the percentage of dead cells was observed. The highest apoptosis index was observed when the MN cells were treated with TGF-β and incubated with the *E. histolytica* [Fig. 2].

MN cells exhibited intracellular Ca^2+^ release. In the presence of TGF-β intracellular Ca^2+^ release was increased, but INF-γ, did not change intracellular Ca^2+^ release [Fig. 3 a and b].

## Discussion

The present study describes IFN-γ and TGF-β levels in the supernatant of MN cells and *E. histolytica* cultures and how these cytokines affect the functional activity of MN cells when in the presence of this parasite. Using an *in vitro* model of amoebiasis we demonstrated that stimulation of MN cells with cytokines decreased the leukophagocytosis, increased the amoebicidal activity and induced death of cells by apoptosis.

Several factors affect cytokine production, especially infections by protozoa [22]. In this study, MN cells and *E. histolytica* cocultures affected the levels of IFN-γ and TGF-β because the presence of virulent *E. histolytica* increased the cytokine levels. A number of studies have shown that virulent *E. histolytica* is associated with the excessive release of proinflammatory cytokines. The supernatant of tissues cultured with *E. histolytica* showed increased cytokine levels in the presence of the virulent strain but not with the non-virulent strain [23]. Cytokines have been shown to exert profound effects on the biological signaling and regulation of important physiological processes [24]. Cytokines may also be related to phagocyte activation and the production of reactive oxygen species [24].

A number of mechanisms possibly contribute to the formation of these reactive oxygen-free radicals. It has been shown that during protozoan infections there is an active participation of the metabolites of oxygen in the production of free radicals [11, 25, 26]. For protection against *E. histolytica* infection, reactive oxygen species [ROS] are important, studies have shown that ROS are able to kill trophozoites and that highly virulent strains are less susceptible to ROS [27, 28].

In the present study, the cytokines tested modulated superoxide release. MN cells increased superoxide release in the presence of *E. histolytica*. Similar results of superoxide anion were obtained during interactions of MN cells and amoeba in the presence of hormones [9], suggesting that these interactions are dependent of immunomodulatory agents.

The functional activity of phagocytes has been assessed during amoeba interactions [9, 28, 29], and phagocytosis has been shown to play an important role in *E. histolytica* pathogenicity. The phagocytic capacity of amoebae are thought to involve leukophagocytosis because amoebae are constantly in contact with leukocytes *in vivo* and must be able to destroy them to survive [9].

The identification of the substances involved in leukocyte activation during amoeba-leukocyte interactions could help to direct the appropriate therapeutic use of these substances in amoebiasis. Here, we evaluated the action of cytokines during leukophagocytosis performed by *E. histolytica* trophozoites. MN cells in the presence of both IFN-γ and TGF-β decreased the cell-ingesting capacity of amoebae.

Phagocytosis and microbicidal activity by phagocytes, with the production of active oxygen metabolites such as free radicals, make up an important defense mechanism against a number of bacterial [8, 10, 12], fungal [30] and protozoal infections [9, 26, 31]. Cytokines such as IFN-γ primarily act on monocytes/macrophages by activating their phagocytic and microbicidal abilities [32].

IFN-γ appears to provide protection against amoebiasis through its ability to activate neutrophils and macrophages to kill the parasite. MN cells stimulated with soluble amoebic extract and IFN-γ have shown an increased production of IFN-γ, associated with reduced diarrhoea in amoebiasis patients [33].

Classical studies involving amoebae and leukocytes [34, 35] have shown that virulent strains of amoebae are lethal to leukocytes, which lose their motility and are then phagocytosed and killed by the amoebae.

Interestingly, in this study, MN cells exposed to *E. histolytica* and treated by cytokines (IFN-γ or TGF-β) showed a decrease in leukophagocytosis but an increase in their amoebicide activity and higher apoptosis index. Studies have related that this increase in microbicidal activity is crucial for the immune response of the host against intestinal amoebiasis [9, 26]. The functional activity modulated by IFN-γ and TGF-β has also been reported for other infections [18].

However, studies support the notion that *Entamoeba histolytica* directly triggers host cell apoptosis on contact and shows that host cell apoptosis facilitates *Entamoeba histolytica* infection in the gut [36]. It is interesting that in this study, the high apoptosis rates were accompanied by a high index of amoeba death after interactions with MN cells stimulated by cytokines.

These results suggest that cytokines plays a beneficial role for the host by activating MN cells against *E. histolytica*, evidenced by higher levels of superoxide and microbicidal activity by MN cells in the presence of IFN-γ or TGF-β.

Cytokine production may be associated with a number of processes such as alterations in intracellular Ca^2+^ by phagocytes [18]. In the present study, TGF-β increased intracellular Ca^2+^ release in MN cells. The literature has demonstrated that increased superoxide release modifies the response of intracellular Ca2+ and the phosphorylation events during oxidative metabolism [37] and in response to hormones [12, 21] and cytokines [18]. It may have a direct effect on MN cells by increasing intracellular calcium release. Here, the increase in superoxide release by MN cells in the presence of cytokines altered the levels of intracellular Ca^2+^ and promoted the amoebicidal activity of these cells.

Considering that this parasite lives in the gut and is in constant contact with mucosal immunological factors and that the TGF-β is an important mediator of mucosal immunity, more studies need to be addressed to clarify the mechanisms involved in host interactions with amoebae.

## Conclusion

In conclusion, the increased death of amoebae during leukophagocytosis suggests that both cytokines [IFN-γ and TGF-β] can modulate the functional activity of MN cells and that these cytokines may play a beneficial role in the control of amoebic infections.

## References

[1] Ximénez CL, Morán P, Rojas L, Valadez A, Gómez A (2009). Reassessment of the epidemiology of amebiasis: state of the art. Infect Genet Evol.

[2] Hewitson JP, Maizels RM (2014). Vaccination against helminthic parasite infections. Expert Rev Vaccines.

[3] Schmid-Hempel P (2008). Parasite immune evasion: a momentous molecular war. Trends Ecol Evol.

[4] Baxt LA, Rastew E, Bracha R, Mirelman D, Singh U (2010). Down regulation of an *Entamoeba histolytica* rhomboid protease reveals roles in regulating parasite adhesion and phagocytosis. Eukaryot Cell.

[5] Honorio-França AC, Launay P, Carneiro-Sampaio MMS, Monteiro RC (2001). Colostral neutrophils express Fc alpha receptors (CD89) lacking gamma chain association and mediate non inflammatory properties of secretory IgA. J Leuk Biol.

[6] Zhang Z, Mahajan S, Zhang X, Stanley SL (2003). Tumor necrosis factor alpha is a key mediator of gut inflammation seen in amebic colitis in human intestine in the SCID mouse-human intestinal xenograft model of disease. Infect Immun.

[7] França-Botelho AC, França EL, Honório-França AC, Gomes MA, Costa-Cruz JM (2006). Phagocytosis of *Giardia lamblia* trophozoites by human colostral leucocytes. Acta Paediatr.

[8] França EL, Morceli G, Fagundes DLG, Rudge MVC, Calderon IMP, Honorio-França AC (2011). Secretory IgA Fcα receptor interaction modulating phagocytosis and microbicidal activity by phagocytes in human colostrum of diabetics. APMIS.

[9] França-Botelho AC, França JL, Oliveira FMS, França EL, Honório-França AC, Caliari MV (2011). Melatonin reduces the severity of experimental amoebiasis. Parasit Vectors.

[10] França EL, Bittencourt RV, Fujimori M, Morais TC, Calderon IMP, Honório-França AC (2011). Human colostral phagocytes eliminate enterotoxigenic *Escherichia coli* opsonized by colostrum supernatant. J Microbiol Immunol Infect.

[11] França EL, Vieira EL, Morceli G, Fagundes DLG, Rudge MVC, Calderon IMP (2012). Transfer of Maternal Immunity to Newborns of Diabetic Mothers. Clin Develop Immunol.

[12] Morceli G, Honório-França AC, Fagundes DLG, Calderon IMP, França EL (2013). Antioxidant Effect of Melatonin on the Functional Activity of Colostral Phagocytes in Diabetic Women. PLoS One.

[13] Seydel KB, Smith SJ, Stanley SL (2000). Innate immunity to amebic liver abscess is dependent on gamma interferon and nitric oxide in a murine model of disease. Infect Immun.

[14] Lotter H, Helk E, Bernin H, Jacobs T, Prehn C, Adamski J (2013). Testosterone Increases Susceptibility to Amebic Liver Abscess in Mice and Mediates Inhibition of IFNγ Secretion in Natural Killer T Cells. PLoS One.

[15] Gordon S, Martinez FO (2010). Alternative activation of macrophages: mechanism and functions. Immunity.

[16] Guo X, Barroso L, Lyerly DM, Petri WA, Houpt ER (2011). CD4+ and CD8+ T cell- and IL-17-mediated Protection against *Entamoeba histolytica* Induced by a Recombinant. Vaccine.

[17] Rafiei A, Ajami A, Hajilooi M, Etemadi A (2009). Th-1/Th-2 cytokine pattern in human amoebic colitis. Pak J Biol Sci.

[18] Fagundes DLG, França EL, Morceli G, Rudge MVC, Calderon IMP, Honorio-França AC (2013). The Role of Cytokines in the Functional Activity of Phagocytes in Blood and Colostrum of Diabetic Mothers. Clin Develop Immunol.

[19] Honorio-Franca AC, Carvalho MP, Isaac L, Trabulsi LR, Carneiro-Sampaio MMS (1997). Colostral mononuclear phagocytes are able to kill enteropathogenic *Escherichia coli* opsonized with colostral IgA. Scand J Immunol.

[20] Pundt N, Peters MA, Wunrau C, Strietholt S, Fehrmann C, Neugebauer K (2009). Susceptibility of rheumatoid arthritis synovial fibroblasts to FasL- and TRAIL-induced apoptosis is cell cycle-dependent. Arthritis Res Ther.

[21] Fagundes DLG, França EL, Hara CCP, Honorio-Franca AC (2012). Immunomodulatory effects of poly (ethylene glycol) microspheres adsorbed with cortisol on activity of colostrum phagocytes. Int J Pharmacol.

[22] Campbell D, Chadee K (1997). Survival strategies of *Entamoeba histolytica*: modulation of cell-mediated immune response. Parasitol Today.

[23] Bansal V, Costantini T, Kroll L, Peterson C, Loomis W, Eliceiri B (2009). Coimbra R. Traumatic brain injury and intestinal dysfunction: uncovering the neuro-enteric axis. J Neurotrauma.

[24] Séguin R, Mann BJ, Keller K, Chadee K (1997). The tumor necrosis factor alpha-stimulating region of galactose-inhibitable lectin of *Entamoeba histolytica* activates gamma interferon-primed macrophages for amebicidal activity mediated by nitric oxide. Infect Immun.

[25] França EL, Feliciano ND, Silva KA, Ferrari CKB, Honório-França AC (2009). Modulatory Role of Melatonin on Superoxide Release by Spleen Macrophages Isolated from Alloxan-Induced Diabetic Rats. Bratisl Med J.

[26] França-Botelho AC, França JL, França EL, Honório-Franca AC, Busatti HGNO, Gomes MA (2010). Relationship between oxidative stress production and virulence capacity of *Entamoeba* strains. J Parasitol.

[27] Richard J, Pearson PJ, Morf L, Singh U (2013). Regulation of H_2_O_2_ Stress-responsive Genes through a Novel Transcription Factor in the Protozoan Pathogen *Entamoeba histolytica*. J Biol Chem.

[28] Ghadirian E, Somerfield SD, Kongshavn PA (1986). Susceptibility of *Entamoeba histolytica* to oxidants. Infect Immun.

[29] Possamai MM, Honorio-França AC, Reinaque APB, França EL, Souto PCS (2013). Brazilian Propolis: A Natural Product That Improved the Fungicidal Activity by Blood Phagocytes. BioMed Res Int.

[30] Casares S, Richie TL (2011). Immune evasion by malaria parasites: a challenge for vaccine development. Curr Opin Immunol.

[31] Pacheco-Yepez J, Jarillo-Luna RA, Gutierrez-Meza M, Abarca-Rojano E, Larsen BA, Rodriguez RC (2014). Peroxynitrite and peroxiredoxin in the pathogenesis of experimental amebic liver abscess. Biomed Res Intern.

[32] Dickson-Gonzalez SM, de Uribe ML, Rodriguez-Morales AJ (2009). Polymorphonuclear neutrophil infiltration intensity as consequence of *Entamoeba histolytica* density in amebic colitis. Surg Infect (Larchmt).

[33] Mortimer L, Chadee K (2010). The immunopathogenesis of *Entamoeba histolytica*. Exp Parasitol.

[34] Lin JY, Seguin R, Keller K, Chadee K (1994). Tumor necrosis factor alpha augments nitric oxide-dependent macrophage cytotoxicity against *Entamoeba histolytica* by enhanced expression of the nitric oxide synthase gene. Infect Immun.

[35] Sanchez-Guillen MC, Perez-Fuentes R, Salgado-Rosas H, Ruiz-Argüelles A, Ackers J, Shire A, Talamas-Rohana P (2002). Differentiation of *Entamoeba histolytica/Entamoeba* dispar by PCR and their correlation with humoral and cellular immunity in individuals with clinical variants of amoebiasis. Am J Trop Med Hyg.

[36] Becker SM, Cho KN, Guo X, Fendig K, Oosman MN, Whitehead R (2010). Epithelial Cell Apoptosis Facilitates *Entamoeba histolytica* Infection in the Gut. Am J Pathol.

[37] Carrichon L, Picciocchi A, Debeurme F, Defendi A, Beaumel S (2011). Characterization of superoxide overproduction by the D-LoopNox4-Nox2 cytochrome b558 in phagocytes-differential sensitivity to calcium and phosphorylation events. Bioch Biophys Acta.

